# Autonomous International Classification of Diseases Coding Using Pretrained Language Models and Advanced Prompt Learning Techniques: Evaluation of an Automated Analysis System Using Medical Text

**DOI:** 10.2196/63020

**Published:** 2025-01-06

**Authors:** Yan Zhuang, Junyan Zhang, Xiuxing Li, Chao Liu, Yue Yu, Wei Dong, Kunlun He

**Affiliations:** 1 Medical Big Data Research Center Chinese PLA General Hospital Beijing China; 2 School of Computer Science & Technology Beijing Institute of Technology Beijing China; 3 Digital Health China Technologies Co Ltd Beijing China; 4 Senior Department of Cardiology The Sixth Medical Center of PLA General Hospital Beijing China

**Keywords:** BERT, bidirectional encoder representations from transformers, pretrained language models, prompt learning, ICD, International Classification of Diseases, cardiovascular disease, few-shot learning, multicenter medical data

## Abstract

**Background:**

Machine learning models can reduce the burden on doctors by converting medical records into International Classification of Diseases (ICD) codes in real time, thereby enhancing the efficiency of diagnosis and treatment. However, it faces challenges such as small datasets, diverse writing styles, unstructured records, and the need for semimanual preprocessing. Existing approaches, such as naive Bayes, Word2Vec, and convolutional neural networks, have limitations in handling missing values and understanding the context of medical texts, leading to a high error rate. We developed a fully automated pipeline based on the Key–bidirectional encoder representations from transformers (BERT) approach and large-scale medical records for continued pretraining, which effectively converts long free text into standard ICD codes. By adjusting parameter settings, such as mixed templates and soft verbalizers, the model can adapt flexibly to different requirements, enabling task-specific prompt learning.

**Objective:**

This study aims to propose a prompt learning real-time framework based on pretrained language models that can automatically label long free-text data with ICD-10 codes for cardiovascular diseases without the need for semiautomatic preprocessing.

**Methods:**

We integrated 4 components into our framework: a medical pretrained BERT, a keyword filtration BERT in a functional order, a fine-tuning phase, and task-specific prompt learning utilizing mixed templates and soft verbalizers. This framework was validated on a multicenter medical dataset for the automated ICD coding of 13 common cardiovascular diseases (584,969 records). Its performance was compared against robustly optimized BERT pretraining approach, extreme language network, and various BERT-based fine-tuning pipelines. Additionally, we evaluated the framework’s performance under different prompt learning and fine-tuning settings. Furthermore, few-shot learning experiments were conducted to assess the feasibility and efficacy of our framework in scenarios involving small- to mid-sized datasets.

**Results:**

Compared with traditional pretraining and fine-tuning pipelines, our approach achieved a higher micro–F1-score of 0.838 and a macro–area under the receiver operating characteristic curve (macro-AUC) of 0.958, which is 10% higher than other methods. Among different prompt learning setups, the combination of mixed templates and soft verbalizers yielded the best performance. Few-shot experiments showed that performance stabilized and the AUC peaked at 500 shots.

**Conclusions:**

These findings underscore the effectiveness and superior performance of prompt learning and fine-tuning for subtasks within pretrained language models in medical practice. Our real-time ICD coding pipeline efficiently converts detailed medical free text into standardized labels, offering promising applications in clinical decision-making. It can assist doctors unfamiliar with the ICD coding system in organizing medical record information, thereby accelerating the medical process and enhancing the efficiency of diagnosis and treatment.

## Introduction

### Background

The *International Classification of Diseases, 10th Revision* (*ICD-10*), is a universally recognized diagnostic categorization system widely used in medical insurance reimbursements, health reporting, mortality assessments, and related fields [1]. The *ICD-10*’s automatic coding mechanism enables rapid and accurate classification and statistical analysis of medical data, offering a scientific foundation for effective hospital administration and decision-making. In addition, the *ICD-10* automatic coding system accelerates disease diagnosis and treatment planning for medical practitioners, thereby improving medical efficacy and quality. Compared with the original *ICD* code, *ICD-10* provides over 14,000 distinct disease codes (in contrast to the thousands in *ICD-9*), enabling more detailed disease classification. This comprehensive system offers clinicians enhanced patient information, supporting the development of more precise treatment plans and care programs, ultimately improving the quality of care and patient satisfaction. Moreover, as an internationally standardized code, *ICD-10* is essential for global public health surveillance, epidemiological research, and international medical cooperation. Consequently, ensuring accurate *ICD* coding remains a critical priority in clinical practice.

In hospital settings, the assignment of *ICD* codes to unstructured clinical narratives in medical records is a manual task performed by skilled medical coders based on the attending physician’s clinical diagnosis. Despite its critical importance, this process is often hindered by inefficiencies such as time consumption, susceptibility to errors, and high costs. Additionally, manual coding cannot always ensure the accuracy of *ICD* codes due to the complexity of code assignment, which requires a thorough consideration of the patient’s overall health condition, including medical history, coexisting conditions, complications, surgical interventions, and specialized diagnostic procedures [2,3].

### Machine Learning Techniques

The need to enhance efficiency and reduce errors has driven the development of various machine learning techniques to automate the medical *ICD* coding process. These techniques can be broadly classified into 4 main categories: rule-based systems [4,5], traditional supervised algorithms [6,7], gate unit–based deep learning approaches [7-9], and pretrained language models (PLMs) [9-19].

First, rule-based systems for automatic *ICD* coding rely on the creation of explicit rules and knowledge bases to map medical records to the appropriate *ICD* codes [4,5]. Although these approaches have been used for decades and have provided a foundation for more advanced techniques, they are limited by their lack of adaptability and scalability.

Second, traditional supervised algorithms, such as gradient-boosted trees, have been utilized for *ICD* coding due to their efficiency in handling large-scale, high-dimensional datasets. These algorithms rely on semistructured preprocessing, which involves organizing and refining semistructured data into a format suitable for analysis [6,7]. For example, Diao et al [6] developed a light gradient boosting machine–based pipeline for automatically coding 168 primary diagnosis *ICD-10* codes from discharge records and procedure texts, achieving an accuracy of 95.2%. Another study integrated long short-term memory networks with attention mechanisms to predict mortality in ICU patients using electronic health records, achieving significantly higher area under the receiver operating characteristic curve (AUC) scores compared with traditional statistical models and stand-alone long short-term memory networks [7].

Third, PLMs are neural network models with fixed architectures trained on large corpora, which can be fine-tuned for specific downstream tasks such as question answering and entity recognition [10-13]. A notable example is bidirectional encoder representations from transformers (BERT), a prominent PLM designed to learn deep bidirectional representations from large-scale unlabeled text data. BERT effectively captures semantic relationships in clinical records and can be easily adapted to various natural language processing (NLP) tasks through task-specific layers [13]. Coutinho and Martins [14] proposed a BERT model with a fine-tuning method for automatic *ICD-10* coding of death certificates based on free-text descriptions and associated documents. Additionally, Yan et al [15] introduced RadBERT, an ensemble model combining BERT-base, Clinical-BERT, the robustly optimized BERT pretraining approach (RoBERTa), and BioMed-RoBERTa adapted for radiology. Liu et al [16] evaluated RadBERT across 3 NLP tasks: abnormal sentence classification, report coding, and report summarization, demonstrating significantly better performance compared with existing transformer language models. Unstructured patient-generated health data can be leveraged to support clinical decision-making, remote monitoring, and self-care, including medication adherence and chronic disease management. By applying named entity recognition and customizable information extraction methods based on medical ontologies, NLP models can extract a wide range of clinical information from unstructured patient-generated health data, even in low-resource settings with limited patient notes or training data [17]. Textual analysis presents numerous opportunities for future medical applications. It can aid in extracting information from various sources of medical data, such as clinical reports, nursing notes, scientific literature, and user-generated content. Additionally, vector-based representation methods can transform textual data within clinical documents into formats suitable for machine learning and can be applied to sequence modeling tasks, including sentiment analysis [18].

Finally, XLNet is another type of PLM that captures both forward and backward contexts of text [19]. It combines the advantages of autoregressive models and autoencoding models while overcoming their limitations. XLNet utilizes a permutation-based objective function that maximizes the expected likelihood of a text across all possible word orderings. It also incorporates the Transformer-XL (Transformer-Extra-Long) architecture, which enables long-term dependency modeling and improved memory efficiency. XLNet has been shown to outperform BERT and other baseline models on several natural language understanding tasks.

### Prompt Engineering Techniques

By contrast, prompt engineering is a technique that involves the careful construction of prompts or inputs for artificial intelligence models to improve their performance on specific tasks. This technique includes selecting appropriate words, phrases, symbols, and formats to guide a large language model in generating high-quality and relevant text. Numerous studies have used prompts for model tuning to bridge the gap between pretraining objectives and downstream tasks, demonstrating that both discrete and continuous prompts can improve performance in few-shot and zero-shot tasks [20,21]. Furthermore, this technique within PLMs has been shown to outperform fine-tuning in various clinical decision-making tasks [22]. It has the advantage of requiring less data and computational resources, making it especially suitable for clinical settings.

There are 2 primary categories of prompting methods: hard prompts and soft prompts [22-25]. Hard prompts involve using an actual text string as the prompt and include methods that automatically search for templates within a discrete space, such as mining-based, paraphrasing-based, and gradient-based approaches [26-28]. The advantages of hard prompts are interpretability, portability, flexibility, and simplicity. However, designing effective prompts for specific tasks requires significant effort and creativity.

Soft prompts, by contrast, are learnable tensors concatenated with the input embeddings and can be optimized for a given dataset. The main advantage of soft prompts is their ability to achieve better performance than hard prompts by adapting to the model and the data. However, they are not human-readable and lack portability across different models.

Prefix tuning and P-tuning are 2 methods of prompt engineering that can enhance performance beyond traditional fine-tuning [22-24]. Prefix tuning is a lightweight approach that keeps the PLM parameters unchanged while optimizing a sequence of task-specific vectors called the prefix [23]. This prefix is added to the input and interacts with the model’s hidden states at each layer. Its success depends on how effectively the prefix is initialized, particularly when data are limited. P-tuning is another prompt tuning strategy that performs comparably to fine-tuning across various tasks [24]. It reduces the number of PLM parameters through self-adaptive pruning and tunes a small number of continuous prompts at the beginning of each transformer layer.

The verbalizer is the final layer that defines the answer space and maps it to the target output. Typically, verbalizers are manually created, which can limit their coverage due to personal vocabulary biases [21,29]. To address this, some studies have proposed automatic verbalizer search methods to identify more effective verbalizers, also known as soft verbalizers [20,30-32].

### Autonomous ICD Coding in Cardiovascular Disease

Cardiovascular disease (CVD) is currently a leading cause of death worldwide, posing a significant risk of mortality among patients [7]. Automatically labeling patients with CVD is essential for clinical decision-making and resource allocation. However, existing prediction models have limitations, including low accuracy, limited generalizability, and an inability to capture multicenter data. To address these challenges, we propose a prompt learning real-time framework based on PLMs that can automatically label long free-text data with *ICD-10* codes for CVDs without the need for semiautomatic preprocessing.

Our framework consists of 4 components: a medically oriented pretrained BERT, a keyword filtration BERT, a fine-tuning phase, and task-specific prompt learning facilitated by mixed templates and soft verbalizers. To validate the efficacy of our framework, we conducted comprehensive evaluations on a Chinese multicenter cardiovascular dataset that includes data from 13,000 patients with CVD. This deliberate choice of dataset ensures the robustness and wide applicability of our framework. We compared our framework with RoBERTa, XLNet, and various BERT-based fine-tuning pipelines to highlight its performance. Additionally, we conducted few-shot experiments to demonstrate its resilience. This work promises to provide valuable insights into enhancing medical knowledge extraction and its effective application, underscoring the need for continued research and development in this promising area. In future work, we plan to implement this fully automated *ICD* coding pipeline across various clinical applications, including clinical decision support systems, cohort studies, and disease early warning and diagnosis systems.

## Methods

### Ethical Considerations

The study was approved by the Ethics Committee of the Chinese PLA General Hospital (S2023-325-02). Ethical approval included a waiver for obtaining informed consent signatures from participants. The study posed no potential harm to participants and did not involve any compensation for their participation. To protect patient privacy, we used regular expressions to parse and redact basic identifying information from the medical records. As these records were created using a standardized template, we ensured that the excerpts extracted for this study did not contain patients’ names.

### Overview

**Figure 1:**
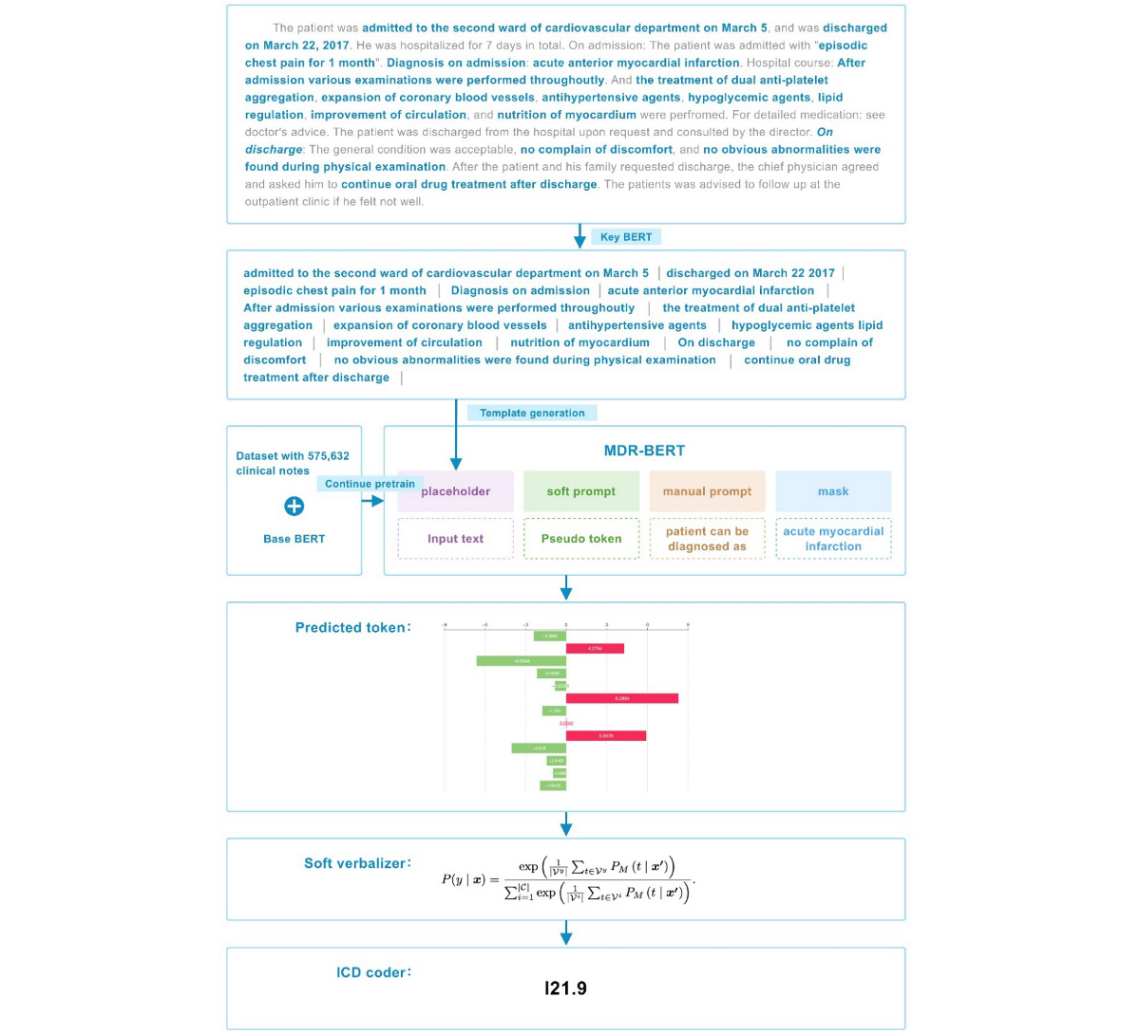
Overall framework of MDR-BERT, Key-BERT, and prompt learning pipeline. BERT: bidirectional encoder representations from transformers; ICD: International Classification of Diseases; MDR: medical domain refinement.

The overall framework of the model is shown in Figure 1. We used a corpus dataset of 575,632 clinical notes to continue training the original BERT model, which we named medical domain refinement-BERT (MDR-BERT), as the PLM for our work. For the classification task, we first applied Key-BERT to filter the discharge summaries. This method extracts keywords and splits long free-text data into shorter sentences.

We then constructed the input template for fine-tuning and prompt learning using 3 components: the soft prompt, the manual prompt, and the mask part. The manual prompt was a handcrafted text prompt containing discrete tokens. The soft prompt was a learnable pseudo-token with a few continuous parameters. The mask part represented the *ICD* coding label. Finally, we used a trainable soft verbalizer to compute and apply the softmax function to the probabilities of the *ICD* classes, producing the output. By designing specific prompts, it is possible to incorporate the knowledge of medical experts into the model, helping it better understand and perform *ICD* coding. These prompts can direct the model to focus on critical sections of the input text, thereby enhancing performance.

### Dataset Characteristics

**Figure 2:**
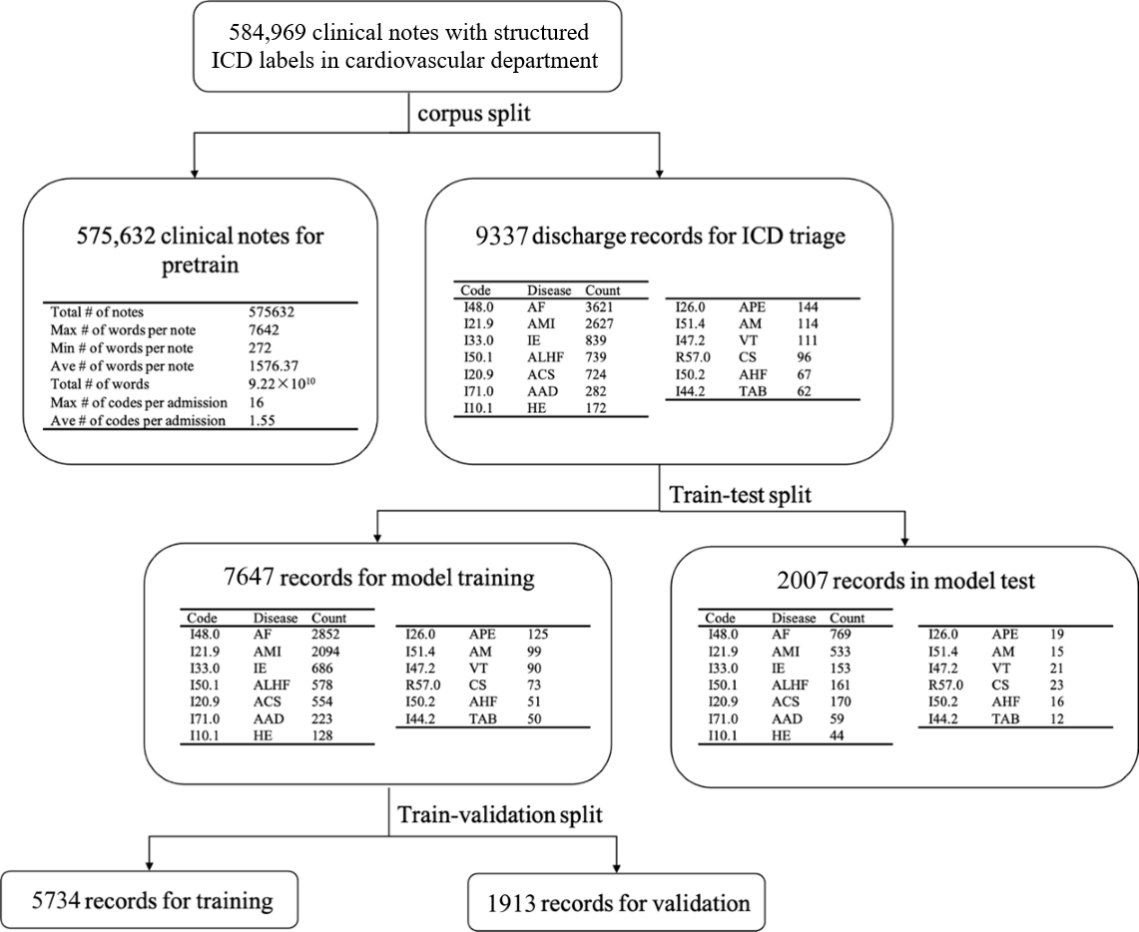
Distribution and basic statistical information of the data set. ICD: International Classification of Diseases.

The cardiovascular dataset used in this study was obtained from the Cardiovascular Department of the Chinese PLA General Hospital’s Medical Big Data Research Center in Beijing, China, which includes 9 medical centers with data aggregated into a comprehensive medical big data platform. Additionally, the hospital is a key center for the treatment of CVDs, with numerous specialized physicians and detailed medical records, making its data highly practical and representative. To ensure privacy, patient names and addresses were desensitized. The data platform consists of electronic health records aggregated from 8 affiliated medical centers. A total of 584,969 clinical notes with structured *ICD* labels were extracted from admission records and discharge summaries in the Cardiovascular Department. We ensured that each diagnosis included at least 50 cases and adopted a stratified sampling approach to divide each disease category into training, validation, and test sets in a 3:1:1 ratio. The detailed distribution and basic statistical information of the dataset are shown in Figure 2.

Based on the long-tailed distribution and clinician selection, 13 diseases were chosen for classification. These diseases include atrial fibrillation, acute myocardial infarction, infective endocarditis, acute left heart failure, acute coronary syndrome (ACS), acute aortic dissection, hypertensive emergency, acute pulmonary embolism, acute myocarditis, ventricular tachycardia, cardiogenic shock, acute heart failure, and third-degree atrioventricular block. The corresponding *ICD-10* codes and abbreviations for these diseases are listed in Table 1. Despite the disparity in the number of cases for different diseases, the imbalance inherent in medical data accurately reflects real-world conditions, taking into account the clinical insights of medical professionals. This imbalance represents the varying frequency at which different diseases occur in clinical practice. By preserving the raw data distribution and avoiding artificial balancing, our training approach aligns more closely with real-world medical practice. As a result, this enhances the model’s generalization ability and its applicability in practical scenarios.

To ensure task independence and prevent data leakage, all clinical notes were divided into 2 parts: the pretraining corpus dataset and the *ICD* coding dataset. The pretraining corpus consisted of a total of 575,632 notes, while the *ICD* coding dataset included 9337 discharge records. The data were stratified by imbalanced *ICD* labels and randomly split into training, validation, and test sets in a 3:1:1 ratio. The sample sizes were as follows: 5734 in the training set, 1913 in the validation set, and 2007 in the test set. We applied regularization to truncate patients’ basic information, as this information could negatively impact the model’s fitting.

**Figure 3:**
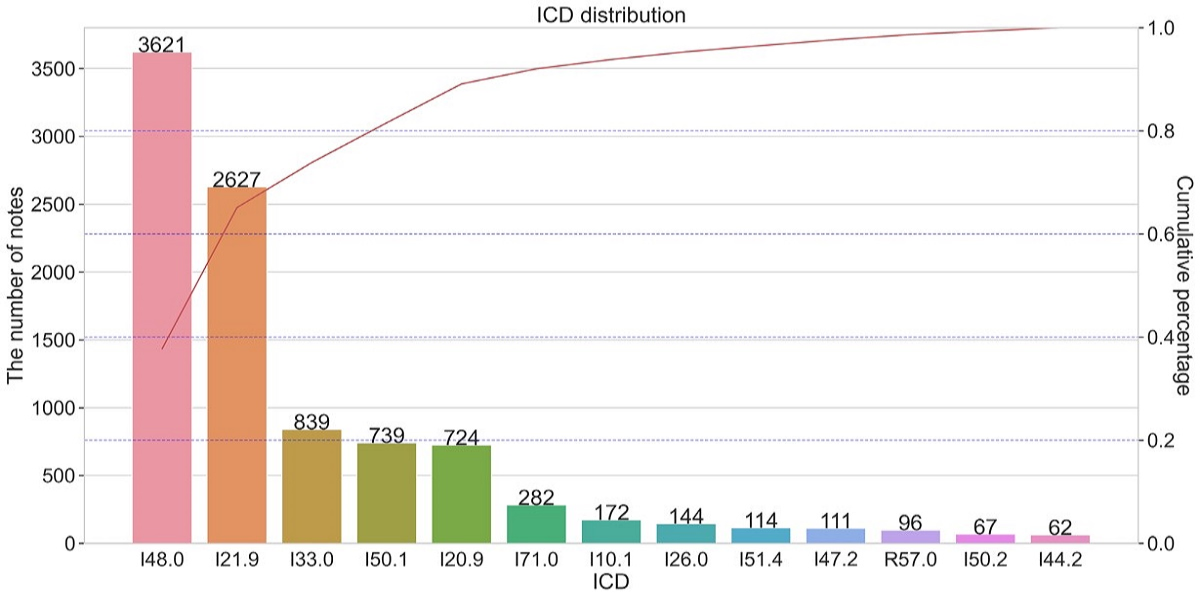
Distribution of ICD codes for the triage task. ICD: International Classification of Diseases.

As shown in Figure 3, the distribution of the 13 *ICD* codes was imbalanced and exhibited a long-tail pattern. The dataset for *ICD* classification contains a total of 4.574 × 10^7^ words, with an average of 490 words per note. The maximum and minimum lengths of the clinical notes are 5243 and 22 words, respectively.

**Table 1:** Overview of target International Classification of Diseases (ICD) codes and disease names.

*International Classification of Diseases* code	Disease (abbreviation)
I48.0	Atrial fibrillation (AF)
I21.9	Acute myocardial infarction (AMI)
I33.0	Infective endocarditis (IE)
I50.1	Acute left heart failure (ALHF)
I20.9	Acute coronary syndrome (ACS)
I71.0	Acute aortic dissection (AAD)
I10.1	Hypertensive emergency (HE)
I26.0	Acute pulmonary embolism (APE)
I51.4	Acute myocarditis (AM)
I47.2	Ventricular tachycardia (VT)
R57.0	Cardiogenic shock (CS)
I50.2	Acute heart failure (AHF)
I44.2	Third-degree atrioventricular block (TAB)

### Pretraining

Our study’s foundational framework is based on BERT, a multilayer bidirectional transformer encoder known for its conceptual simplicity and empirical effectiveness [33]. This architecture consists of 12 layers, a hidden size dimension of 768, and 12 self-attention heads [13]. BERT’s inherent self-attention mechanism provides the versatility to handle various downstream tasks by allowing the interchange of relevant inputs and outputs, making it well-suited for our task involving *ICD* classification through clinical records.

To adapt BERT to the specific requirements of our task, we continued training the PLM using an extensive medical corpus, resulting in MDR-BERT. During the tuning process, we selected a batch size of 32, considering the constraint of a maximum sequence length of 512 tokens. The Adam optimization algorithm was used with a conservative learning rate of 2 × 10^–5^. The training was carried out over 15 epochs, an empirically determined figure based on the characteristics of the clinical dataset.

### Key-BERT

The Key-BERT method offers a novel self-supervised framework for extracting keywords and keyphrases from textual content using deep learning techniques [34]. This approach leverages the contextual and semantic features provided by bidirectional transformers, with a particular focus on the influential BERT model. The method’s architecture is designed for end-to-end training, utilizing a contextually self-annotated corpus that enables the model to develop a nuanced understanding of the complex relationships between words and their semantic meanings. In the *ICD* coding task, Key-BERT leverages BERT’s context-aware capabilities to extract keywords from the document, quickly identify the sections relevant to *ICD* coding, and reduce the risk of miscoding caused by misinterpreting or overlooking critical information in the text.

A distinctive feature of Key-BERT lies in its automated keyword labeling process. This process effectively utilizes contextual insights from bidirectional transformers to construct a carefully curated ground truth dataset. This approach bypasses the labor-intensive task of manual labeling and eliminates the need for domain-specific expertise.

The repository of self-labeled data generated by Key-BERT is partially shared with the NLP community, contributing to a deeper and more comprehensive understanding of keyword extraction techniques across various domains. This collaborative effort enhances the landscape of knowledge and expertise, driving advancements in the field of NLP and semantic information extraction.

To extract keywords using Key-BERT, the contextual feature vector for each word in a sentence is obtained by passing the sentence through the pretrained BERT model. Let *S* = [*w_1_*, *w_2_*, ..., *w_n_*] be a sentence consisting of *n* words, where *w_i_* is the *i*th word in the sentence and *E_i_* is the contextual feature vector of the *i*th word in the sentence. The sentence embedding vector, denoted as *E_s_*, is obtained by averaging the feature vectors of all the words in the sentence:

E_i_ = BERT_Embedding(w_i_) **(1)**

E_s_ = (E_1_ + E_2_ + ···+ E_n_)/(n) **(2)**

The cosine similarity metric is used to measure the similarity between the sentence embedding vector and the feature vectors of candidate keywords or keyphrases.

Cos_SIM(E_i_, E_s_) = (E_i_ × E_s_)/(||E_i_|| × ||E_s_||) **(3)**

The top-scoring keywords or keyphrases are returned as the most relevant to the document. Additionally, key medical terms are directly extracted using the medical diagnostic table, ensuring that essential terminology is accurately identified and applied.

### Fine-Tuning and Prompt Learning

To fully leverage the clinical knowledge embedded within the dataset, our fine-tuning approach mirrors the unsupervised task used in the initial pretraining phase, known as masked language modeling (MLM). MLM involves randomly masking a predetermined proportion of input tokens, and the model then attempts to predict these masked tokens based on context. This process, commonly called a Cloze task, helps the model learn contextual relationships effectively.

For the fine-tuning phase in this study, we maintained the MLM framework to align with the pretraining procedure. A consistent masking rate of 15% was applied across the dataset. In addition to the fine-tuning process, we introduced prompt learning during parameter tuning. This approach involved the construction of a template comprising 4 distinct components: the input text, a soft prompt, a manual prompt, and a masking component. The manual prompt included discrete tokens that reflected the downstream task expected by the PLM. By contrast, the soft prompt comprised trainable continuous vectors, which enhanced the model’s adaptability.

Formally, automatic *ICD* coding, as a text multiclassification task, can be denoted as (*x*, *y*), where *x* is the set of discharge summaries and *y* is the *ICD* code set of the 17 chosen discharge diagnoses as labels. Given a clinical record *x* ∈ *X*, it can be annotated with *ICD* codes of discharge diagnosis *y^x^* ∈ *Y* and a sequence of discrete input tokens *x* = (*x*_0_, *x*_1_, ..., *x_k_*), where *k* is the number of tokens in the sequence. Prompt learning can be achieved via modifying the *x* to a prompt format *x* = fp(*x*), where the template f_p_(·) will insert a number of extra embeddings to *x* along with a masked token, denoted by <[MASK]>. Compared with hard prompts, soft prompts replace some fixed manual components with trainable embeddings (continuous vectors) of the same dimension as the PLM. After that, *x* is fed into *M*, to predict the masked token, which is in accordance with the objective of *M*. The output of *M* will be a distribution over the fixed vocabulary *V* of *M*. The next crucial step is to map tokens in *V* to *y* for the downstream task with a mapping 
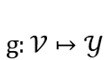
, known as verbalization. In a word, there are 2 essential components to be studied, the template of prompt *x*′ = f_p_(x) and the mapping of verbalizer 
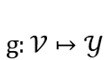
.

A mixed template of prompts in this paper is used. For simplicity, the prompt function *x*′ = f_p_(x) is denoted as a sequence template:

x′ = [P_0_, P_1_, …, P_j_], x, [P_j__+1_, P_j__+2_, …, P_t_], [MASK] **(4)**

where *P_i_* refers to the *i*th token in the template and *t* is the number of prompt tokens beyond *x*. *P_i_* does not necessarily meet *P_i_* ∈ *V* other than manual hard prompt. As *x*′ is fed to the PLM, the prompt tokens are also mapped to the embedding space, where we can assume that the tokens denoted as <[soft]> in the template can be tuned during training as pseudo-tokens. A simple example of a prompt template for automatic *ICD* coding could be as generated as follows:

x′ = <x><[soft]>be encoded as <[MASK]> **(5)**

Once these templates were formulated, the model inputs, along with the established templates, were processed through the trainable MDR-BERT model. Notably, in the final layer of the most advanced pipeline, a soft verbalizer mode was used. This mode manages the mapping process between the predicted tokens and the final *ICD* codes. The innovative feature of the soft verbalizer is its substitution of tokens in the verbalizer with trainable vectors, each tailored to a specific class. Generally, the verbalizer maps the probabilities of infrequent words in the vocabulary to the probabilities of the labels. The set of label words is denoted as *V*, the label space is *Y*, and *V_y_* represents the subset of label words for label *y*. The final estimation of the probability for label *y* is calculated using equation 5, where *g* is utilized to convert the probability of label words to the probability of the label:

*P*(*y*|*x*___*_P_*) = *g*(*P_M_*([MASK] = *v*|*X*___*_P_*)|*v* ∈ *V_y_*) **(6)**

This strategy enhances the precision and semantic accuracy of the generated outputs, enabling a more precise alignment between predicted tokens and the definitive *ICD* codes. Consequently, it is unnecessary to manually build an explicit mapping 
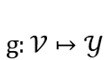
. for the soft verbalizer, as the trainable vectors do not have explainable semantic meaning. A matrix operator can represent the soft verbalizer as 
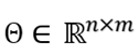
 [22-25], where *n* represents the size of *y* and *m* represents the dimension of output embeddings from *M*. For the verbalizer, *θ_i_* denotes the *i*th row of 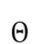 as the trainable vector of the *i*th class. The soft verbalizer replaces the original decoder head of *M* by mapping the embeddings of *x′* from *M*, denoted as *e*(*x′*), to the distribution over the classes of *y*. We denote the resulting mapping from 
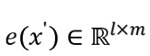
 to the prediction of the embedding of <[MASK]> as 
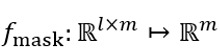
, where *l* is the sequence length of *x′*. And then, the probability of class *y* can be calculated as follows:



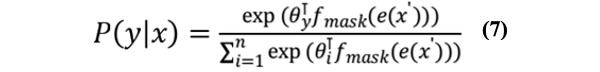



The loss from the automatic *ICD* coding task can be backpropagated to tune only the embeddings for the prompt template and the verbalizer. The loss function can be expressed as follows:



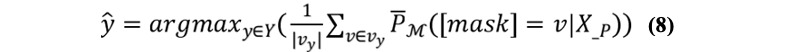



Ultimately, the model learns to generate and map the most appropriate *ICD* codes to the corresponding discharge record.

The experiments were conducted using the OpenPrompt framework [22-25]. For prompt learning, we utilized the Adafactor optimizer for soft and mixed prompt templates, while the AdamW optimizer was used for the PLMs and soft verbalizers. In conventional fine-tuning, we applied the AdamW optimizer to the MLP heads and PLMs. To expedite the experiments, we used 2 Nvidia TESLA V100 GPUs, each with 16-GB memory, and set the batch size to 32 due to memory constraints.

The model’s performance is influenced by variations in hyperparameters. In the comparisons presented, hyperparameters were carefully optimized for each model. To determine the optimal configuration, we used a random search strategy. This approach involves generating multiple random combinations of parameters, evaluating the performance of each combination, and selecting the one that yields the best results. Accuracy and AUC were chosen as the primary optimization objectives during the random search, as they intuitively reflect the model’s classification performance. The strategy involved 100 training runs, each using randomly generated hyperparameters from the defined search space. To effectively address model overfitting, we carefully adjusted the dropout rate within a range of 0.1-0.5. After numerous training iterations, we found that the optimal dropout rate for the prompt learning phase is 0.382, while for the prompt tuning phase, it is 0.1563. In the prompt learning phase, a higher dropout rate contributes to improved generalization, serving as an effective safeguard against overfitting. In the subsequent fine-tuning phase, a lower dropout rate is used to ensure the model retains its learned attributes while enabling further performance enhancement. The optimal hyperparameters for the models are detailed in Table 2.

**Table 2:** The optimal hyperparameters and their search space.

Hyperparameters	Search space	Optimal hyperparameter	
		Prompt learning	Fine-tuning
Learning rate	log.uniform [1*10-5, 3*10-1]	0.0048	0.0121
Batch size	4	4	4
Gradient accumulation steps	range[2,10]	4	3
Dropout	range[0.1,0.5]	0.382	0.1563
Optimizer	[adamw, adafactor]	adamw	adafactor
Prompt learning rate	log.uniform[1*10-5, 3*10-1]	0.3	—^a^
Verbalizer learning rate	log.uniform[1*10-5, 1*10-1]	0.007	—

^a^Not available.

### Evaluation Metrics

To thoroughly evaluate and compare the performance of the models, we used a range of metrics, including micro–*F*_1_-score, macro-AUC, and accuracy. The definitions for micro-averaged precision and micro–*F*_1_-score are provided in equations 9-11, while the macro-AUC is defined in equations 12 and 13.



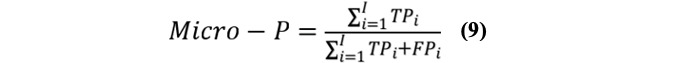





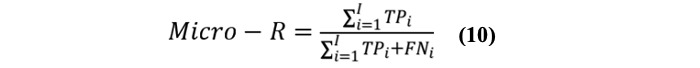



Micro–*F*_1_-score = [2 × (micro-*P*) × (micro-*R*)]/[(micro-*P*) + (micro-*R*)] **(11)**

where TP*_i_*, FP*_i_*, and FN*_i_* represent true positives (correctly assigned instances), false positives (incorrect assignments by automated methods), and false negatives (correct instances omitted by automated methods), respectively, of code *i*, and *l* is the size of the sample space. The micro–*F*_1_-score is the harmonic mean of micro-*P* and micro-*R*, and a bigger value of micro–*F*_1_-score indicates a better performance.



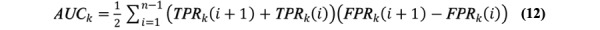





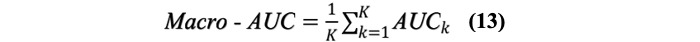



where n is the number of thresholds and K is the number of classes.

### Data and Code Availability

Data acquisition requests can be made by contacting the corresponding author (KH). Given the sensitive nature of the hospital data, it cannot be released publicly. However, part of the downstream subtask data is currently undergoing desensitization and approval processes. The source code for this study is publicly available on GitHub [35].

## Results

### Performance of Different Pipelines

To evaluate the performance of different methods, we implemented 4 state-of-the-art algorithms: BERT [15], XLNet [18], RoBERTa [19,36], and prompt learning [22]. These PLMs were integrated with various algorithms to create 6 main pipelines: BERT with fine-tuning, XLNet with fine-tuning, RoBERTa with fine-tuning, BERT with prompt learning, MDR-BERT with prompt learning, and MDR-BERT with both fine-tuning and prompt learning. MDR-BERT is a PLM developed by further pretraining BERT on our medical corpus.

**Figure 4:**
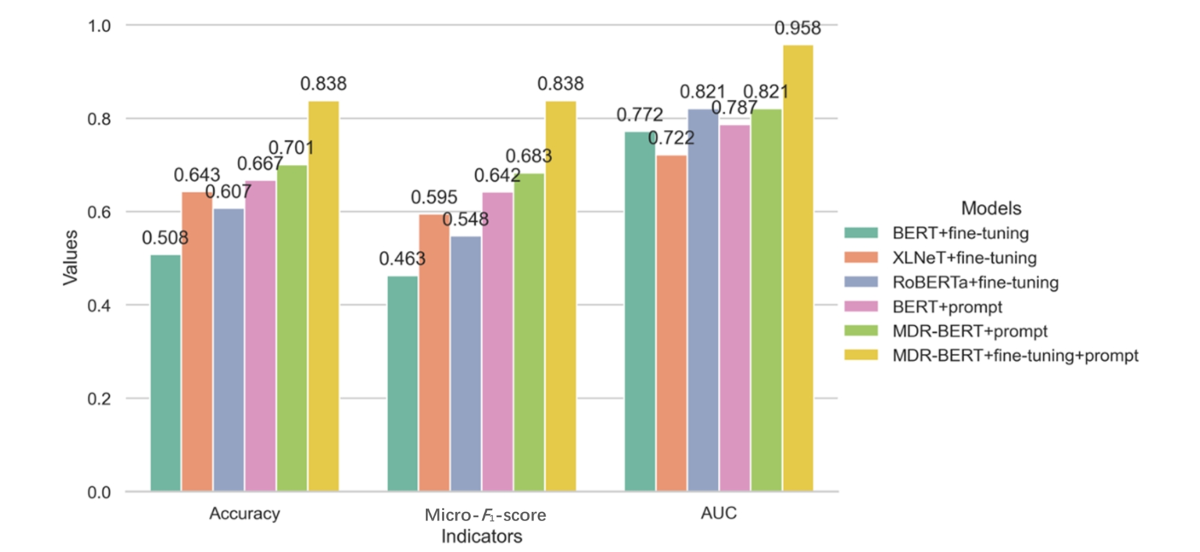
The optimal hyperparameters and their search space. AUC: area under the receiver operating characteristic curve; BERT: bidirectional encoder representations from transformers; MDR: medical domain refinement; RoBERTa: robustly optimized BERT pretraining approach; XLNet: extreme language network.

As shown in Figure 4, MDR-BERT with fine-tuning and prompt learning achieved the highest performance across all evaluation metrics, with a micro–*F*_1_-score of 0.838, a macro-AUC of 0.958, and an accuracy of 0.838. MDR-BERT with prompt learning alone performed slightly worse than the combined fine-tuning and prompt learning approach, but both outperformed the other pipelines by a significant margin. This suggests that continued pretraining on clinical records can significantly enhance the performance of the PLM for the task, while freezing parameters may hinder the adaptation of smaller PLMs to the task.

Among the other pipelines, BERT with prompt learning achieved the highest accuracy (0.67) and the highest micro–*F*_1_-score (0.64), though its macro-AUC (0.79) was slightly lower than that of RoBERTa with fine-tuning. This suggests that prompt learning, as a lightweight tuning approach, can match or even surpass traditional fine-tuning methods, aligning with the findings of Taylor et al [22].

We also conducted a comparison with state-of-the-art methods and selected 2 prominent models: mt5-xxl (11B) and Qwen2.5-72B-Instruct. Among these, mt5-xxl demonstrated the best performance in text classification, while Qwen2.5-72B-Instruct excelled as a large language model. For mt5-xxl, we fine-tuned the model using the training and validation sets from our fine-tuning dataset, setting the “prefix_text” to “Classify the following text:”. For Qwen2.5-72B-Instruct, we conducted experiments using both zero-shot and retrieval augmented generation methods. In the zero-shot setting, we used prompts to constrain the diagnostic scope, allowing the model to make inferences based on the input information. For the retrieval augmented generation approach, we first encoded the training set using BGE-M3 (BAAI general embedding multilinguality, multigranularity, and multifunctionality) and stored it in a Faiss vector database. During the testing phase, we retrieved cases and classification results relevant to the input content and concatenated them with the prompt to enhance model performance.

**Figure 5:**
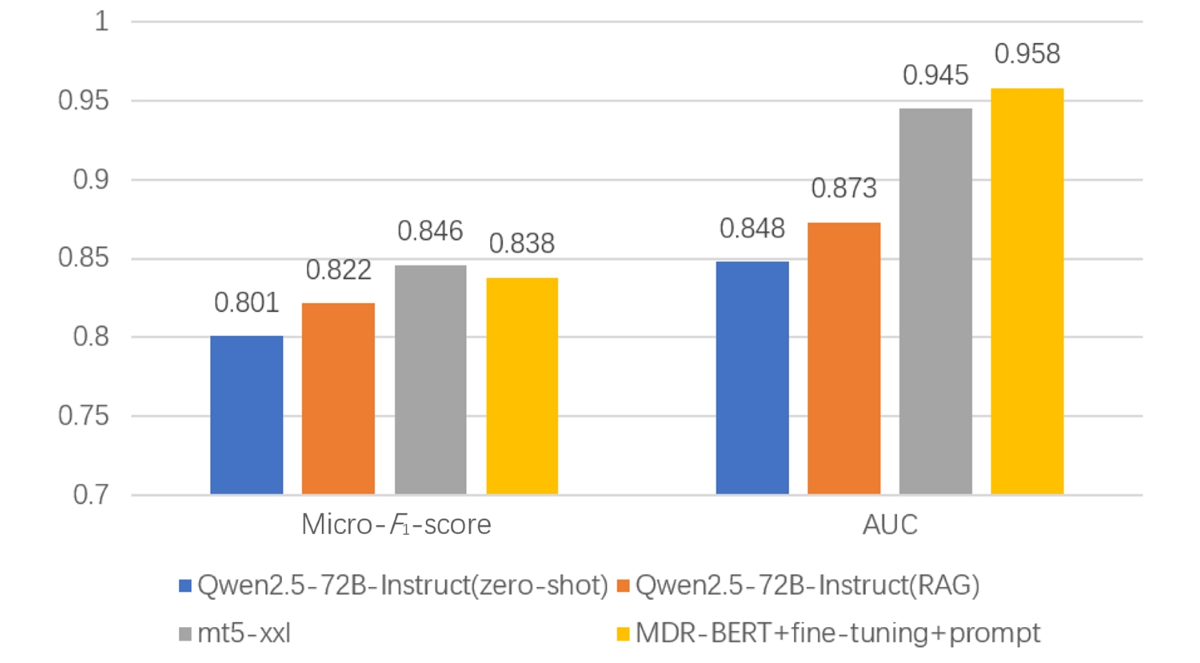
Micro-*F*_1_-score and AUC values for the MDR-BERT model versus the QWEN2.5 and mt5-xxl models. AUC: area under the receiver operating characteristic curve; MDR: medical domain refinement; BERT: bidirectional encoder representations from transformers.

The experimental results indicate that the micro–*F*_1_-score for the mt5-xxl method is 0.846, and the AUC value is 0.945. In comparison, the micro–*F*_1_-score for the Qwen2.5-72B-Instruct method was 0.822, and the AUC value was 0.848. However, the accuracy of both methods does not surpass that of our MDR-BERT model (Figure 5). After a series of strategic optimizations, our MDR-BERT model achieved results comparable to the fine-tuned mt5-xxl on specific tasks. This is primarily due to the specific structure of the medical records, which can be effectively captured by models with fewer parameters, meaning that overly complex models are not necessary to achieve good performance.

### Performance of Different Prompt Learning Modes

We evaluated the performance of MDR-BERT under various settings of prompt learning and fine-tuning, using 3 types of templates (manual, soft, and mixed) and 2 types of verbalizers (manual and soft) as hyperparameters.

**Figure 6:**
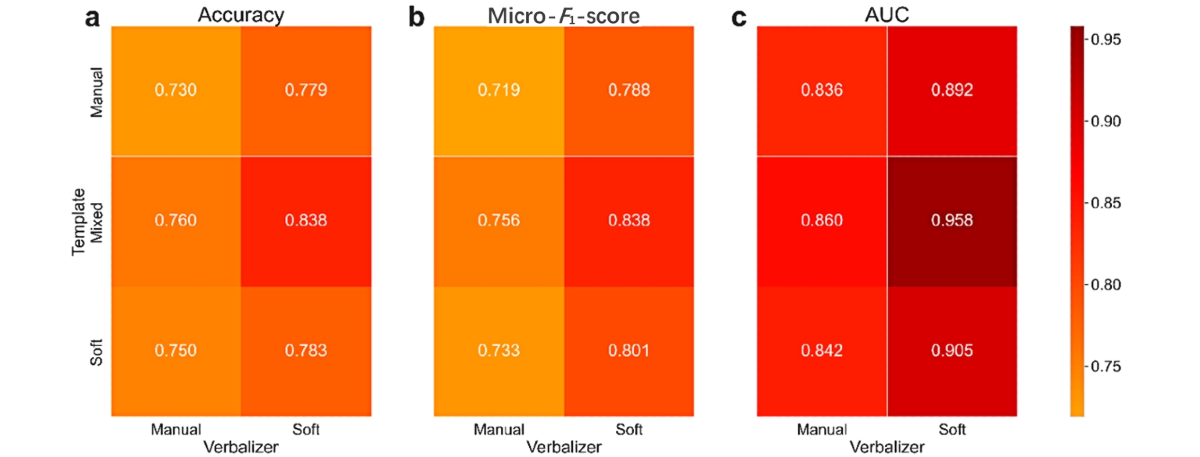
Comparison among different prompt combinations in verbalizer and template. AUC: area under the receiver operating characteristic curve.

For templates, both scripted and self-adaptive patterns performed well independently, and their combination had a cumulative positive effect on performance. For verbalizers, the self-adaptive type outperformed the traditional manual vectors and had a greater impact on overall performance. As shown in Figure 6, the combination of mixed templates and the soft verbalizer achieved the best results.

Take the following prompt template as an example:

Mixed template: {“placeholder”: “text_a”} patient {“soft”:“ can be diagnosed as ”} {“mask”}.

For the following case:

The patient was discovered to have bradycardia and unconscious disturbance 7 days ago as a result of physical examination. After consultation with the director, lipid-lowering drugs were added. No diarrhea was detected, and no medication was administered at home. Permanent cardiac pacemaker implantation under local anesthesia was carried out, and after the surgery, cephalosporin for injection was utilized to prevent infection.

The classification result by our model is as follows: “The patient can be diagnosed as {third-degree atrioventricular block}.”

For the mixed template, the patient’s bradycardia requires management through the implantation of a permanent pacemaker, indicating that bradycardia is a major medical concern. By applying soft verbalizers, we can guide the correct diagnosis by emphasizing both the reason for the pacemaker implantation and the underlying cause of bradycardia: “The patient can be diagnosed with third-degree atrioventricular block.”

### Performance of MDR-BERT With Fine-Tuning and Prompt Learning

**Figure 7:**
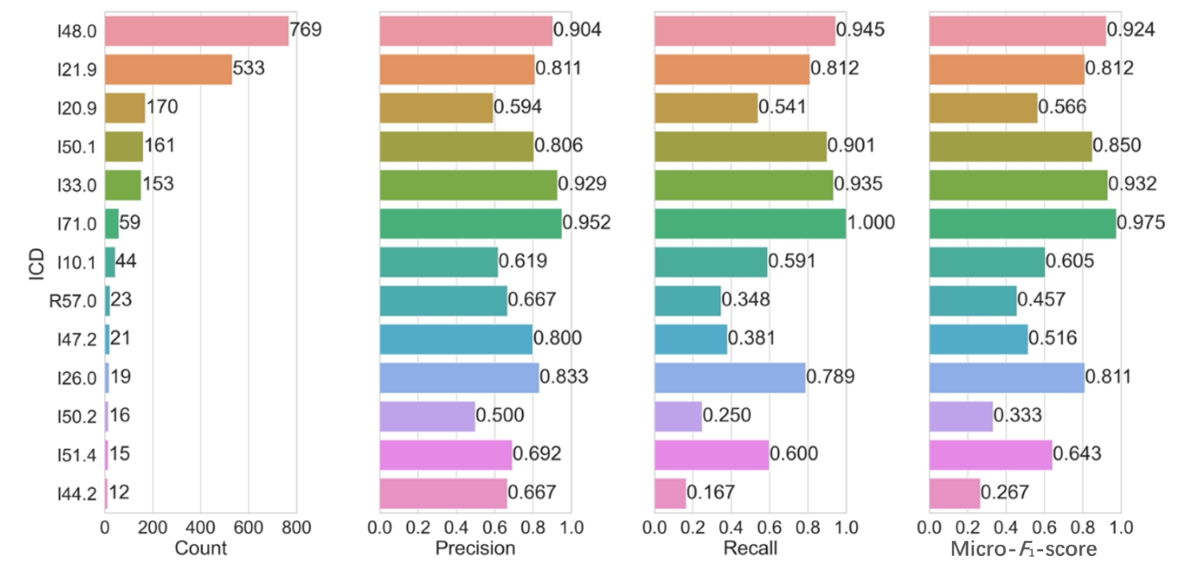
Precision, recall, and micro-*F*_1_ scores of every ICD code in the MDR-BERT pipeline with fine-tuning and prompt learning. BERT: bidirectional encoder representations from transformers; ICD: International Classification of Diseases; MDR: medical domain refinement.

We evaluated the performance of the MDR-BERT pipeline, incorporating both fine-tuning and prompt learning, for each ICD code using precision, recall, and micro–*F*_1_-score. Figure 7 presents the results for these metrics across the 13 ICD classes.

The pipeline achieved high scores for most *ICD* codes, although the scores varied depending on the data distribution and sample size for each code. We observed a weak positive correlation between sample size and model performance, suggesting that larger samples enhanced the model’s learning capability. Conversely, smaller samples tended to have lower micro–*F*_1_-scores, with a trade-off between precision and recall for certain classes. Although our prediction accuracy for ACSs is relatively low, further analysis revealed that in actual clinical settings, ACS was frequently misdiagnosed as cardiac edema (hypertensive emergency) and pulmonary embolism (acute pulmonary embolism). These diseases exhibit similar clinical manifestations and, therefore, require meticulous differential diagnosis to rule out other possibilities. We believe that the overlap of symptoms is a major cause of the difficulty in classifying the model and that inconsistencies in medical histories recorded by physicians further complicate the model’s ability to differentiate similar pathologies. Despite these variations, our pipeline demonstrated satisfactory performance across the different *ICD* codes.

### Few-Shot Learning

**Figure 8:**
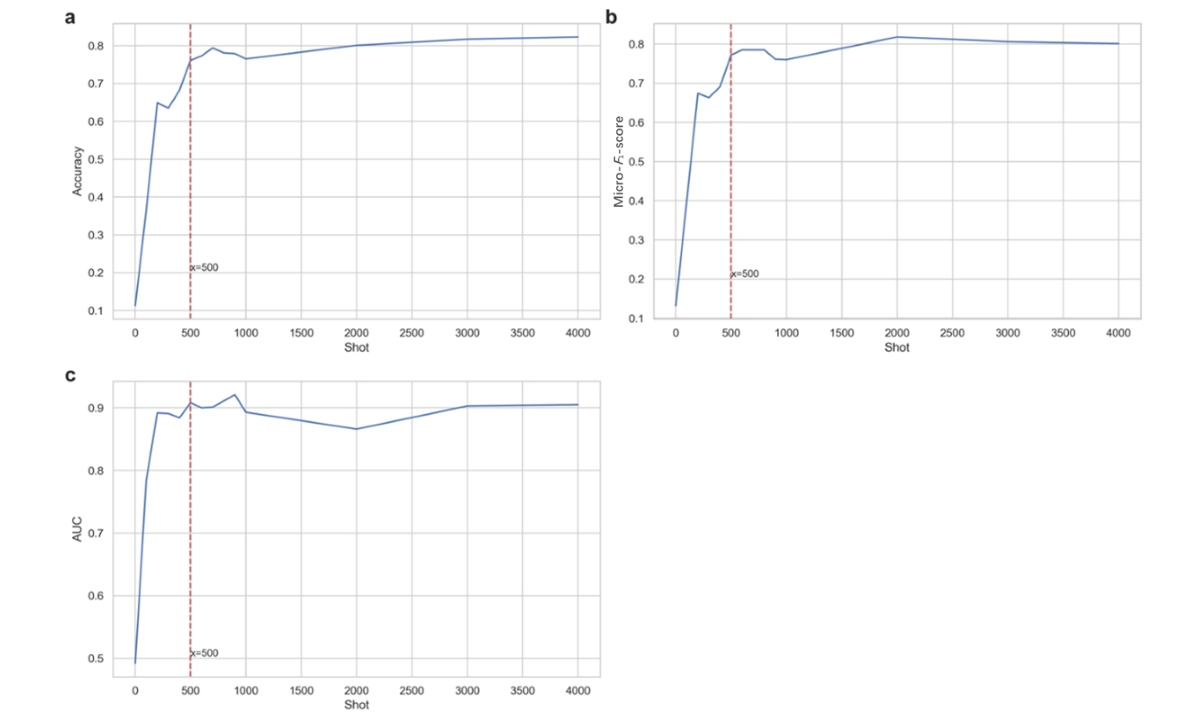
Few shots experiments on MDR-BERT with fine-tuning and prompt learning. AUC: area under the receiver operating characteristic curve; BERT: bidirectional encoder representations from transformers; MDR: medical domain refinement.

We conducted few-shot experiments to evaluate the performance of the fine-tuned MDR-BERT with the prompt learning pipeline using different sample sizes from the training set. We randomly selected samples ranging from 1 to 4000 and evaluated the models on the test set. Figure 8 shows the accuracy, micro–*F*_1_-score, and macro-AUC scores for each sample size.

The objective of small-sample learning is to develop models that can learn effectively and make accurate predictions with only a small number of samples, such as 500 or fewer. As shown in Figure 8, when the sample size reaches 500, the model’s accuracy, AUC score, and other indicators not only achieve relatively high scores but also reach an inflection point and plateau. At this point, the model produces a relatively satisfactory outcome. This indicates that for the task of *ICD* coding using medical records, 500 samples may be sufficient for the model to learn the key features needed to distinguish between different diagnoses. It suggests that the model has captured enough information to make effective predictions. Additionally, the workload involved in annotating 500 medical texts is manageable and feasible. This number strikes a balance between the effort required for data preparation and the performance gains achieved by the model. Given the complexity and specialized nature of medical records, annotating 500 examples provides a comprehensive representation of the dataset while staying within practical limits. This makes it a reasonable and efficient choice for training the model to achieve satisfactory performance in *ICD* coding tasks.

## Discussion

### Principal Findings

An automated *ICD* coding system for long free-text data is a fundamental platform for clinical research and practice, including clinical trials and pharmacoeconomic management. In this study, we developed a framework based on Key-BERT, a continuously trained and tunable PLM, combined with task-specific prompt learning. We collected a total of 584,969 clinical notes from admission records and discharge summaries in the cardiovascular departments of 8 medical centers.

We used most of the data to continue pretraining a medical corpus and used an independent set of 9337 discharge records with 13 *ICD* codes for CVDs in the *ICD* classification subtask. Although the MDR-BERT model has some limitations, such as restricted generalization capacity and constraints on the length of context it can effectively process, it is important to note that medical texts often have a consistent structure and are generally less dependent on extensive contextual information. Given these characteristics of medical literature, our model is designed to avoid the errors commonly associated with the inherent limitations of BERT’s methodology. The structured nature of medical documents enables the MDR-BERT model to function effectively within its designed parameters, mitigating potential issues that could arise from the broader weaknesses of the BERT framework when applied to more contextually complex or varied text types. To remove irrelevant information and limit the input token size, we filtered and truncated all the data for the *ICD* task into keyword-based segments using Key-BERT. The data were then stratified and split into training, validation, and test sets, with the test set used independently for final evaluation.

This study primarily focused on transformer-based algorithms, which have been widely applied and shown superior performance in large-scale medical long free-text tasks [4,11,16,17]. These algorithms can leverage PLMs that capture the semantic and syntactic information of natural language from extensive corpora, leading to significant performance improvements through multicenter datasets.

We compared 6 pipelines for the classification downstream task: BERT with fine-tuning, XLNet with fine-tuning, RoBERTa with fine-tuning, frozen BERT with prompt learning, frozen MDR-BERT with prompt learning, and tunable MDR-BERT with prompt learning. The prompt learning setup included 3 types of templates and 2 types of verbalizers. Among these pipelines, MDR-BERT with fine-tuning and prompt learning achieved the best performance on the test set, attaining a micro–*F*_1_-score of 0.838, a macro-AUC of 0.958, and an accuracy of 0.838.

Compared with the pretraining models of RoBERTa and XLNet, our model achieved superior performance in terms of final accuracy and micro–*F*_1_-score. This improvement was primarily due to the targeted optimization of the methods and the medical data we selected, which substantially enhanced the model’s performance. Although RoBERTa and XLNet have larger pretraining corpora compared with BERT, our approach benefited more from using a continuation training corpus built from real electronic health records. This specialized data, tailored to our specific requirements, provided a greater enhancement to the model than more general pretraining data. This is why MDR-BERT performs comparably to, or better than, these alternatives in our settings. The favorable outcome of this pipeline can be attributed to the use of a large-scale corpus-based PLM and the task-specific enhancements from the combination of fine-tuning and prompt learning [16,20,22-25]. Fine-tuning acts as a model adapter, aligning the model distribution with the task distribution and addressing domain shift and task mismatch issues inherent in PLMs. Prompt learning, with its compact prefix representation and sparse attention mechanism, augments the training data with diverse and natural examples. This augmentation helps mitigate data scarcity and label noise issues in small-sized datasets for downstream tasks.

The combination of fine-tuning and prompt learning acts as a regularization term that balances model complexity with data quality, ultimately enhancing overall performance. This integrated approach highlights the potential of leveraging advanced transformer-based models and customized learning strategies to improve automated medical coding and other clinical tasks.

Among the different prompt learning setups, the mixed template and soft verbalizer achieved the best performance. The soft template method outperformed the manual templates method, which can be attributed to the greater semantic and syntactic information, broader search space, and reduced trial-and-error process associated with the soft template method, making it more effective and less time-consuming [23,24].

The mixed template method is a hybrid approach that combines the advantages of both soft and manual templates. It uses a manual template as a base prompt to provide human-readable instructions and natural language labels, while a soft template serves as an auxiliary prompt to provide tunable embeddings that can adapt to specific downstream tasks. This way, the manual template leverages existing knowledge, while the soft template enhances expressiveness and flexibility.

For the verbalizer, the self-adaptive type had a significantly greater impact on overall performance compared with traditional manual vectors. The soft verbalizer adjusts to the optimal label space for each task and the scale of the pretrained model, rather than being limited by a fixed set of tokens [22,24]. This enhances the accuracy and robustness of the predictions, as well as the diversity and naturalness of the labels. Additionally, by tuning the verbalizer alongside other continuous prompts, it retains the benefits of prompt tuning over fine-tuning, eliminating the need to maintain a separate copy of model parameters for each task during inference.

To explore the influence of sample size on the performance of our pipeline, we conducted few-shot experiments with a range from 1 to 4000 shots. The results showed unsatisfactory evaluation metrics for small-scale shots, but performance improved rapidly and stabilized at around 500 shots. This suggests that for mid-sized language models, such as BERT, the semantic understanding and representation capabilities may not be strong enough. Therefore, tuning the parameters of the PLM with an appropriate sample size is necessary to achieve better performance on specific tasks.

Our research confirms that *ICD* classification tasks can be effectively accomplished by continuously optimizing the BERT model. Although this study used cardiology data for training, our model development strategy is not limited to this specific dataset; substituting the training data with data from other departments would also yield the expected outcomes. Therefore, our model demonstrates remarkable generalization capability. We firmly believe that the model we have developed, combined with the expertise of professional physicians, can effectively address the challenges of *ICD* classification for various diseases.

### Limitations

Despite the reasonable performance of our pipeline, this study has certain limitations. First, we trained both the corpus part and the classification task of the framework solely in the cardiovascular department. As a result, the conclusions of this paper may not be generalizable to other medical fields. Second, the *ICD* classification subtask only involved 13 CVD codes, which is not comprehensive enough for clinical practice. Future research could expand to explore the automatic encoding of additional critical heart diseases or even extend to the entire clinical field. This could potentially enhance the applicability and effectiveness of the proposed approach for a broader range of clinical tasks. Third, our model aims to establish an automated analysis system using medical text. However, medical data are inherently multimodal, and modality augmentation can lead to improvements in accuracy. In this context, models such as label alignment for multimodal prompt learning [37] and multimodal equivalent transformer [38] are designed to handle multimodal data, demonstrating the greater potential for future advancements.

### Conclusions

We proposed a real-time framework for *ICD* coding from long medical field–related text to *ICD* labels, eliminating the need for semistructured preprocessing. This framework incorporates Key-BERT, a continuously trained and tunable PLM, and task-specific prompt learning with mixed templates and soft verbalizers. We evaluated our model on a multicenter cardiovascular dataset and applied it to predict 13 *ICD* codes for CVDs, achieving high performance. Our model also demonstrated transferability and generalization across different centers.

Furthermore, we conducted few-shot experiments to investigate the impact of data size on model performance. The results showed that while the framework was effective on smaller datasets, a certain sample size was necessary to achieve a relatively stable performance level. This study serves as a benchmark for exploring the feasibility and performance of prompt learning in the subtask of large language models or PLMs. Using a multicenter dataset, the approach demonstrated robust performance across hospitals, highlighting its potential for broad deployment.

Few-shot learning experiments demonstrated feasibility with small-scale datasets, enabling applications for local training on single centers or various single-disease databases. The real-time model identifies *ICD* codes directly, accelerating automated coding compared with semiautomatic approaches that require segment preprocessing. This is particularly impactful for clinical decision support systems that rely on real-time *ICD* coding data.

Overall, the prompt learning paradigm achieved cutting-edge *ICD* assignment accuracy while offering deployability, few-shot learning capacity, and low latency—advantages that are highly beneficial for health care applications. This automated *ICD* coding pipeline could be further implemented in various clinical applications, such as clinical decision support systems, cohort studies, and disease early warning and diagnosis systems.

## Abbreviations

ACS: acute coronary syndrome
AUC: area under the receiver operating characteristic curve
BERT: bidirectional encoder representations from transformers
BGE-M3: BAAI general embedding multilinguality, multigranularity, and multifunctionality
CVD: cardiovascular disease
ICD: International Classification of Diseases
MDR: medical domain refinement
MLM: masked language modeling
NLP: natural language processing
PLM: pretrained language model
RoBERTa: robustly optimized BERT pretraining approach
Transformer-XL: Transformer-Extra-Long
XLNet: extreme language network
